# Primary care for people with Parkinson's disease in Brazil: A referral flowchart based on risk of falls

**DOI:** 10.3389/fpubh.2022.836633

**Published:** 2022-07-25

**Authors:** Rafaela Simon Myra, Micheline Henrique Araújo da Luz Koerich, Elaine Cristina Gregório, Alessandra Swarowsky

**Affiliations:** ^1^Brazilian Parkinson's Disease Rehabilitation Initiative (BPaRkI), Center for Health and Sports Sciences (CEFID), Santa Catarina State University (UDESC), Florianópolis, Brazil; ^2^Center for Health and Sports Sciences (CEFID), Santa Catarina State University (UDESC), Florianópolis, Brazil; ^3^Physical Therapy Program, AdventHealth University, Orlando, FL, United States

**Keywords:** Parkinson's disease, workflow, community health planning, primary health care, South America, Brazil

## Abstract

**Background:**

People with Parkinson's disease (PD) need to exercise to have a better quality of life. The risk of falling needs to be considered when choosing and implementing exercise interventions. Flowcharts are used to facilitate referrals in Brazilian primary care network, but there is no specific one for PD.

**Aim:**

To develop a referral flowchart for people with PD in Brazilian primary care based on the risk of falls and scientific evidence in the context of a multidisciplinary approach.

**Methods:**

The development of the referral flowchart was accomplished in three steps; (1) relevant literature was reviewed (2) semi-structured interviews (in focus groups) were conducted with primary health care professionals to investigate the current care for people with Parkinson's disease, and (3) the information obtained from the previous steps were analyzed to inform the development of the referral flowchart.

**Results:**

The fall risk-based flowchart uses the 3-step-fall-prediction tool. The primary health care professional should refer the person with a low risk of falls to activities with minimal supervision and those with a higher risk of falls to specialized neurology services. Neurology services are also the referral target for persons presenting significant mobility restrictions (i.e., restricted to a wheelchair or bed). The referral occurs according to what is available in Brazilian primary care.

**Conclusion:**

This flowchart might be the first step to build a multidisciplinary approach for people with Parkinson's disease in Brazilian primary care. The next stage of this study is the validation and subsequent implementation of the flowchart through the primary care at Unified Health System in Brazil.

## Background

The progressive nature of Parkinson's disease (PD) and the range of symptoms accompanying PD is a complex challenge for the Health System to manage successfully (1–3). As PD progresses, falling can present as an early symptom, and it becomes increasingly frequent in advanced PD, as is the presence of dementia and severe dysphagia (4).

Fall often have deleterious consequences for people with PD, their caregiver, and the quality of life of both (1–3). Falls and fear of falling in PD can lead to (1) significant limitations to daily-life activities (5); (2) pain and decreased independence (6); (3) upper and lower limb fractures (7); (4) increased morbidity (1). The most common causes of hospitalization in people with PD are pneumonia, motor decline, urinary tract infection, and hip fractures. Also, increasing age and presence of dementia were commonly associated with increased mortality (8).With that information we can assume the relationship between the falls and the increase in mortality (1).

Many hospitalizations and attending primary medical care following a fall will have a significant cost impact on the health system (9). Such cost impact may be minimized if the primary care (PC) pathway for prevention and management of falls in PD people was integrated and standardized (10). Fall prevention programs have also been carried out in some other alterations, such as diabetes (11), diabetic neuropathy (12), osteoporosis (13), elderly people (14), and it obtained satisfactory results. Integrated models of multidisciplinary care have been proposed to improve the quality of care for people with PD (15, 16). These types of integrated models could be adapted for low to middle-income countries (16). Brazil has a well-established Unified Health System (UHS), which offers an integrated care model, especially in PC (17). PC comprises the Family Health Strategy (FHS) with allied-health professionals' teams who work collaboratively to develop and deliver care plans that best suit each patient, the presenting symptoms and achieve the expected patient outcomes (17).

Even with an integrated model of care, the Brazilian PC UHS does not have a standardized approach for preventing, treating, and managing falls in people with PD. Brazil primary care is generalist and focused on prevalent diseases, which means that some diseases do not receive as much attention and care. Recent data show that 57% of the people with PD from different regions of Brazil, in general, were not prescribed physiotherapy, and 75% did not do other therapeutic activities, such as speech therapy, occupational therapy, and psychology treatment. This fact may be related to the lack of information and experience in treating PD (18). Despite evidence that exercise reduces falls in people with PD (19, 20), referred patients encountered a variable and *ad-hoc* approach to falls' care and management, with minimal multidisciplinary care provided (21). This situation can be explained by the lack of an explicit policy for referrals and subsequent fall management in the Brazilian PC UHS.

Given the increasing life expectancy of Brazilians (22), the number of people presenting with PD tends to increase, resulting in higher demand for care. As falls represent a significant determinant of quality of life, our study aimed to create a referral flowchart based on the fall risk in people with PD to guide the PC's health professionals.

## Methods

The flowchart development occurred in three steps: (1) literature review, (2) focus group interviews with professionals from the Family Health Strategy (FHS) and (3) refinement of the flowchart elements. The Ethics Committee for Human Beings (CESPH) of the State University of Santa Catarina approved this study under number 2,950,524.

### Literature review

A detailed non-systematic literature review sought the highest level of evidence specific to PD (Guidelines, Meta-analyzes, Systematic Reviews, and Randomized Controlled Trials). We searched for information regarding the recommended treatment according to disease severity, the occurrence of falls, and fall risk. We used the following databases - PubMed, SciELO, Lilacs, Medline, Cinahal, PEDRO - with the keywords Parkinson's disease AND falling OR fall OR falls. We included articles in English and Portuguese without the restriction of date.

### Focus groups interviews

The study was conducted at the Health Centers (HC) of the four health districts (Center, Continent, North, and South) of Florianópolis - Santa Catarina, Brazil, that offered PC services. There are 45 HC spread across the health districts and 125 FHS teams working in them. The FHS teams, work in the HC and are the first contact with people with PD arriving at PC.

The FHS teams are formed primarily by a general practitioner, or specialist in Family Health, or a Family and Community physician; generalist nurse or specialist in Family Health; nursing assistant or technician; and community health agents. It is relevant to say that rehabilitation professionals work in the HC but don't integrate the FHS teams and people who need the rehabilitation service must be referred by FHS professionals.

Florianópolis is the capital city of Santa Catarina State and is the second largest in the number of inhabitants, with approximately 508,826 individuals (22). Also, it is the Brazilian reference (model) for structuring the UHS due to excellent health indicators and population coverage of 90% (23).

Before recruiting FHS teams between January 2016 and December 2017, a survey of people diagnosed with PD was carried out through a municipality's information system management (GeI - Geinfo / DIPLAN). A list of people with PD who engaged in PC services was used to determine those health units with the highest PD caseloads. The FHS were recruited by e-mail, and the focus groups (FG) were formed.

The Consolidated criteria for the REporting Qualitative research Checklist (COREQ) were used to guide FG interviews. The COREQ is a 32-item checklist to assist researchers in reporting essential aspects of the research team, study methods, context of the study, findings, analysis, and interpretations (24). We conducted the FG interview with the FHS teams to determine how they approach and manage people with PD at the PC. It includes the opinions of professionals on the role of PC in the treatment of people with PD and the main barriers and strengths faced in their care. We also examined existing specialized services (polyclinics and rehabilitation centers) and identified the services offered in HC (walking, physical activity, and nutrition groups) to propose the referral flowchart.

After agreeing to participate, the FG meetings were scheduled and held in the HC of each district. A total of eight focus groups took place between January and June 2019, conducted by a moderator, member of the research team, who took detailed interview notes. The FG health professionals number ranged from two to nine, and the interviews lasted from 20 min to 40 min (average of 30 min). There were no conflicts of interest between the researchers and FGs.

A semi-structured interview guided the questions and was recorded by audio. Researchers identified possible central themes, phrases, and impressions. The following issues were framed into questions that each FG discussed: (I) Experience with PD; (II) Diagnosis and Management for people with PD, (III) Referrals, (IV) Specialized Care; and (V) Options for multi-professional activities available at the health unit. Two researchers read the transcribed data and created codes using open coding to themes and subthemes. When differences in interpretation occurred, a third researcher was consulted, and the majority view was prevailing. After the eighth FG, no new information and themes emerged, and we identified the saturation point of the interviews. To assist the organization of the collected data, we used the software package ATLAS.ti8.

### Flowchart elaboration

The data obtained from the qualitative research was used to build and scaffold the referral flowchart based on the risk of falls for people with PD. In addition, the services that were available in PC in the city of Florianopolis and the data from the literature review were incorporated and assisted in the development, structure, and sequencing of the flowchart. The proposal for structuring the PC services for people with PD was structured into: (1) **PD care recommendations – Crucial points based on the literature review** and (2) **The referral flowchart based on the risk of falls - How does it work?**

## Results

The results are organized in three steps: (1) Literature review results: in this section, we will aboard the PD care recommendations and show the crucial points for treating people with and without risk of falling. (2) Focus group results: Here we present the qualitative data and its contribution to the construction of the flowchart. (3) The referral flowchart structure and how to employ it: in this section, we present the creative process of the flowchart and a manual for using it.

### PD care recommendations—crucial points based on the literature review

PD is a multisystemic disease with several motor and non-motor symptoms. The drug treatment available is only partially effective, requiring multidisciplinary care to complete the approach. The involvement of professionals such as physiotherapists, speech therapists, nurses, psychologists, nutritionists, among others, is as significant as the physician's attention (21). The European Physiotherapy Guideline for Parkinson's Disease (2014) states that ideally, a neurologist and a nurse (or other professional with experience in managing PD to access the disease) should always be involved in treating people with PD, corroborating with different specialties to improve their physical well-being (25).

Worldwide, several guidelines recommend multidisciplinary care for the treatment of persons with PD. These include the guidelines produced by the Dutch National Professional Allied Health Associations on speech therapy, occupational therapy, and nutrition (26–30). In addition, a multidisciplinary approach is considered more favorable for PD managing motor and non-motor symptoms (31).

Currently, it is recognized that when performing an exercise, people with PD benefit in different physical (32, 33) and mental aspects (34). Recent evidence also suggests that aerobic exercise may slow the progression of PD symptoms (35). However, special care is required to prescribe exercise for people with PD, accounting for the ' 'individual's mobility status, as well as motor and non-motor impairments. People at risk of falling should be observed more carefully to avoid accidents.

The Brazilian UHS recognizes health as a right for the people and a duty for the state (36). The government must ensure it through social and economic politics, reduce disease risks, and give equal access to health services (36). Specifically, for neurological diseases, in 2005, the Ministry of Health created the National Policy for Attention to Patients with Neurological Disease targeted at people with these health conditions (37). In 2017, the Clinical Protocol and Therapeutic Guidelines for Parkinson's Disease were approved. It aimed to structure the care network, define care services, and establish flowcharts for people with PD. However, it emphasized only clinical aspects of the disease-related medication and surgery, leaving aside other health-related therapies and practices, including physical therapy or any multidisciplinary referral services. In addition, to date, the implementation of this Protocol is still being structured. Thus, it is noted that PD care in the Brazilian UHS presents opportunities for improvement (38).

### Approach to people with PD—with or without risk of falling

When taking the clinical decision about exercise prescription and the need for referring people with PD with accuracy, it is necessary to consider the high rate of falls in this population. PD falls are heterogeneous, recurrent, causing morbidity (1), and might be present since the early stages of the disease (2, 3). Falls can lead to activity limitations (5), pain (6), decreased independence, fear of falling (39), injuries, disabling fractures, among others (5).

Falls may be associated with generic (aging-related) or PD-related factors (21). Generic risk factors include advanced age, polypharmacy and sedative use, cardiac arrhythmia, hypotension, depression, female gender, osteoporosis, weakness due to inactivity, use of visual aids and, anxiety. PD-related risk factors include: (1) disease severity, (2) axial stiffness, (3) cognitive impairment, (4) dyskinesias, (5) history of falls, (6) abnormal posture, (7) high dose of levodopa, (8) use of dopamine agonists, (9) reduced mobility (10) postural instability, (11) freezing of gait (FOG) or festination, and (12) urinary incontinence (21, 40). Environmental risk factors (quality of sidewalks and streets, lack of home adaptations, type of footwear used) also contribute to the high rate of falls (40).

Although falls can occur in the early stages of the disease, falls are not frequent, with a low level of association with the person's daily activities (19). With disease progression, the number of falls increases due to postural and gait changes while the people are still active (19). Although people with PD have an increased risk of falls in the later stages, they are less mobile, so the number of falls tends to be lower (19). Therefore, falls and their complications need to be considered when choosing the best treatment. And as mentioned earlier, physical exercise and physical therapy are essential for improving the quality of life of people with PD (19, 25, 32, 33, 41, 42).

Currently, several tools help to predict falls in PD. Scales as *Functional Gait Assessment* (37, 43) and *Mini-Balance Evaluation Systems Test* (44) are well structured but are lengthy and challenging to be applied by the primary health care network health professional who is unlikely to be a physiotherapist. An alternative is the 3-step fall prediction test, which assesses falls in the past year (Yes/No), freezing of gait in the past month (Yes/No), and comfortable gait speed (Yes/No) (Table 1) (45–47).

**Table 1:** Subjects and answers of the qualitative interviews.

**Subjects discussed with the teams**	**Most mentioned answers**
Experience with PD	- Low experience in treating PD
Diagnosis and Management for people with PD	- No recognition of clinical signs and symptoms of PD; - Lack of experience in making the disease diagnosis.
Referrals	- Lack of PD referral protocols - The teams refer people with PD to multidisciplinary programs available in the HC; - Some teams to multidisciplinary services outside the HC, - Most teams are not aware of multidisciplinary services outside the HC
Specialized Care	- The main approach of the teams is to referring people with PD to the neurologist's assessment.
Options for multi-professional activities available at the health unit	- Activities usually indicated are physical exercise, pain support, psychological support, nutrition groups, dance groups, walking groups, speech therapy

*Elaborated by the authors*.

The 3-step-fall-prediction test is objective and accessible to daily use, and it is validated for people with PD (46, 47). It determines the probability of falling in the next 6 months and rates it as a low (17%), moderate (51%), or high (85%) risk of falls, based on the weighted sum of scores for the 3 questions. (46). The test requires periodic reevaluations (in six months) and is crucial for establishing care routines, (45, 46). Although no training is required for physical therapists, it is recommended that other health professionals learn how to measure gait speed.

The 3-step-fall prediction test has been externally validated in 171 PD people showing accuracy to discriminate fallers from non-fallers (AUC = 0.83; 95% CI 0.76–0.89) (46). The test has recently been administered using a self-report measure for gait speed with similar accuracy to the original 3-step clinical prediction tool (47). The self-reported 3-step-fall prediction test may be useful to identify people with PD at risk of falls in e/tele-health settings (47), or to use in HC with no space to test for gait speed and then referral to other services accordingly.

Those people with PD who are at low risk of falls could engage in group activities available at the PC and require less attention from the health professional. On the other hand, fallers require more outstanding care and may be referred to rehabilitation services such as Specialized Rehabilitation Centers (RECs). Another critical point to remember is the need for more focused attention for lower-mobility people. Those who are mobility-restricted and recurrent fallers also need to be referred to RECs for treatment.

### Qualitative data and its contribution to the flowchart

Approximately 120 e-mails were sent to the coordinators of HC in Florianópolis. Six HC declined to participate, and ten HC agreed. Two were excluded from the final analysis due to unpredicted events occurring on the spot (lack of professionals), totaling eight HC and eight FHS interviewed. We interviewed 37 FHS professionals-−12 community health agents, 10 nurses, 10 physicians, two nurse residents, two medical residents, and one dentist.

The results of the interviews presented in Table 1 and it shows that professionals have little experience in treating people with PD. In their view, referring people with PD to the neurologist's assessment (specialized care) should be one of the main approaches. However, despite knowing the referral protocol, most have never done it, as they claim not to receive high demand from people with PD who seek the service for specific disease treatment. Also, the lack of PD referral protocols for other services is one of the barriers in caring for these people at this level of care.

Medical practitioners report that the correct diagnosis of PD is an essential requirement for appropriate treatment. But, as it is a specific condition and not so frequent (or not correctly identified by them), they report a lack of experience in making the diagnosis. They prefer to refer them to neurologists. Thus, the proposed flowchart is intended to assist professionals with PD cases already diagnosed and confirmed by specialized neurologists. In addition, the lack of experience of these professionals also hampers the recognition of clinical signs and symptoms (such as the frequency of falls) that can be used to indicate/recommend activities and trigger referral to other professionals (such as physiotherapists and physical educator professionals)

FHS professionals recognize the need for multidisciplinary care and, likewise, the need to refer people with PD to other professionals and activities offered by PC. When they do, the activities usually indicated are physical exercise, pain support, psychological support, nutrition groups, dance groups, walking groups, speech therapy, among others that occur within the HC. Usually, these groups are carried out by professionals in the indicated area. Some teams also refer people with PD to multidisciplinary services outside the HC, although most report not being aware of these services.

Some professionals interviewed also believe that their role in treating people with PD is related to support / adjustment of medications and providing documents (medical certificates, expert / judicial reports, request for exams, and consultations). In their perception, most people with PD use the service for renewing medical prescriptions for their disease control, failing to other possible treatments. In this way, the flowchart can help professionals visualize what is available in the primary health care network and quickly refer people with PD to multidisciplinary services.

### The referral flowchart based on the risk of falls—how does it work?

**Figure 1:**
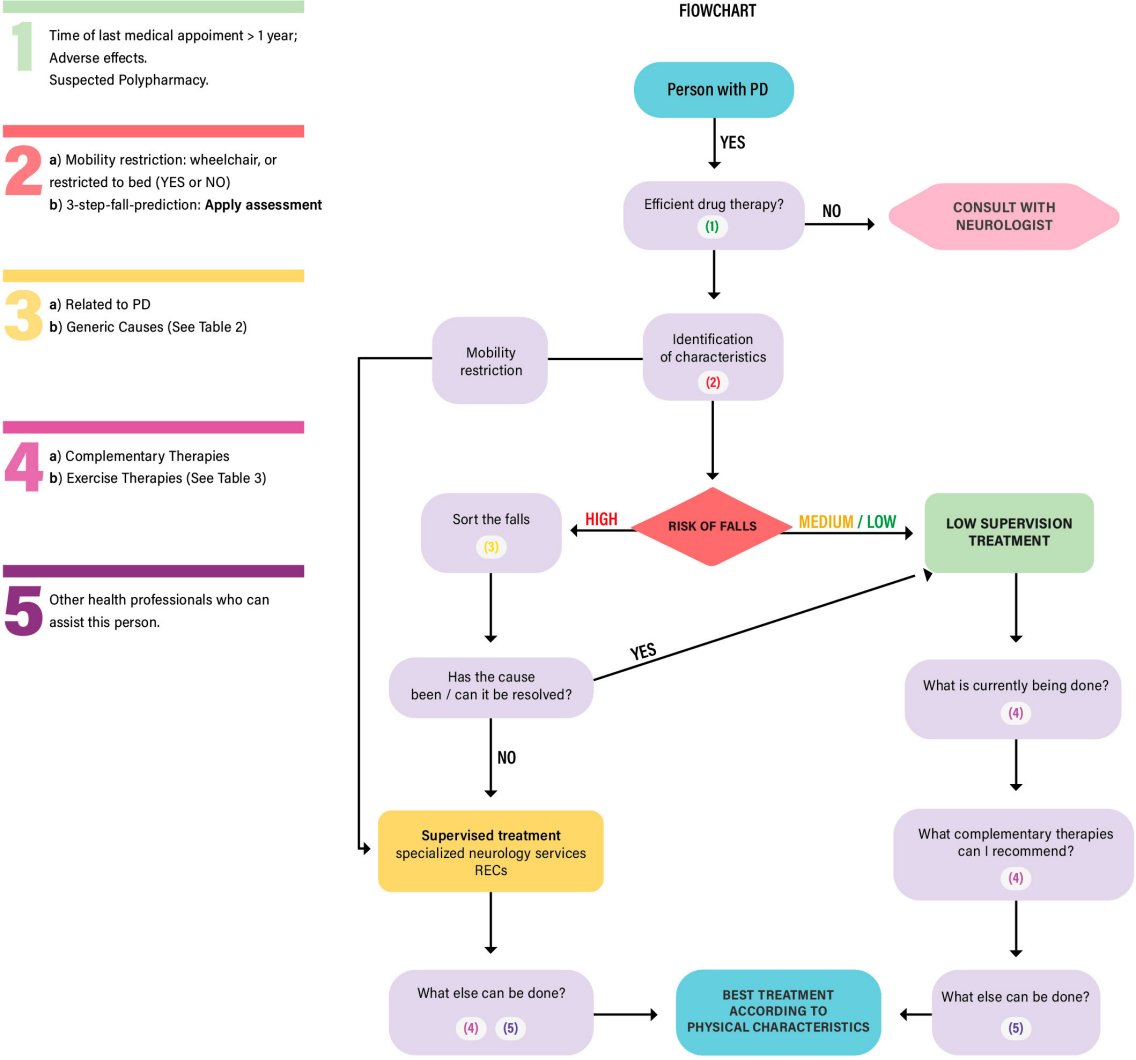
Elaborated by the authors. RECs: Specialized Rehabilitation Centers. PD: Parkinson's Disease.

Flowcharts are practical tools for patient assessment, maintenance, and management; thus, they assist the health professional with the management and care proposal (involving risk and vulnerability assessment) (48) This flowchart is a suggestion to direct the FHS teams in the PC and enhance the quality of care offered to people with PD (Figure 1).

The following tables must be used within the referral flowchart. The Table 2 is a 3-step-fall-prediction test (46, 47). This test is crucial for performing the flowchart. The evaluator should take the measurements, mark which option the person fits, and then continue with the flowchart.

**Table 2:** 3-Step-fall-prediction.

**Assessing the probability of falling in People with Parkinson's Disease**				**Score**
Step 1 Ask your patient Have you fallen in the past 12 months? Yes = 6 No = 0				
Step 2 Ask your patient Have you experienced freezing of gait in the past month? Yes = 3 No = 0				
Step 3 Time you patient walking over the middle 4 m of 6 m walkway at their self-selected comfortable pace: >3.6 s to walk 4m = “Yes” Yes = 2 No = 0				
			Total Score	
Total Score	0	2–6	8–11	
Probability of falling in next six moths	Low (17%)	Moderate (51%)	High (85%)	
Tick appropriate box	□	□	□	

*Table elaborated based on: (46) Paul SS, Canning CG, Sherrington C, Lord SR, Close JCT, Fung VSC (2013) Three simple clinical tests to accurately predict falls in people with Parkinson's disease. Mov Disord. 28:655–62; (47) Almeida RSA, Piemonte MEP, Cavalcanti HM, Canning CG, Paul SS (2021) A self-reported clinical tool predicts falls in people with Parkinson's Disease. Mov Disord. 8:427–34. M, meters*.

After performing the test, and in case of falls, the health professionals should go to Table 3, which refers to PD's fall classification (40), based on modifiable and non-modifiable factors. Modifiable factors may be related to an inappropriate environment - for example, the person's type of shoe can be switched to a safer option that avoids falls. Another example is the high dosage of levodopa or polypharmacy. In this case, the person can be referred to a pharmacist or neurologist to update the medication. Non-modifiable factors may be related to genetic factors (gender), disease severity, among others (40). From that table, the evaluator can check the possible reasons for the falls, manage them, or refer to specialized neurology services, which offer a supervised physiotherapy approach.

**Table 3:** Fall classification in Parkinson's disease.

**Falls classification**	
**Generic - aging-related**	**Related to PD**
Advanced age Female Gender Sedative medication Polypharmacy (use of > 3 drugs other than PD specific) Postural hypotension, orthostatic syncope, autonomic dysfunction Cardiac arrhythmia Arthrosis Use of an assistive device Weakness due to inactivity Visual and ocular motor impairment Daily use of alcohol Other comorbidities (dizziness, peripheral neuropathy) Environmental Hazards (At home and in the community; inadequate lighting, throw rugs, slippery floors…) Osteoporosis Depression	Fall history Disease severity PD medication (Higher doses of levodopa/ use of dopamine agonists) Slow mobility Shuffling and slightly scaled gait FOG and festination Postural instability Transfers Cognitive Impairment Axial Rigidity Dyskinesias Urinary Incontinence Dual Tasking

*Table elaborated based on: (40) van der Marck MA, Klok MPC, Okun MS, Giladi N, Munneke M, Bloem BR (2016) Consensus-based clinical practice recommendations for the examination and management of falls in patients with o disease. Parkinsonism Relat Disord 20, 360–9. PD, 'Parkinson's Disease; FOG, Freezing of Gait*.

Table 4 refers to complimentary and exercise therapies indicated for people with PD. (31, 40).

**Table 4:** Complementary and exercise therapy for people with PD.

**Complementary therapies**	**Exercise therapy**
Speech therapy Psychological treatment Nutritional accompaniment	Physiotherapy – group Activities available in the HC (Yoga, Tai Chi, dance classes, walking groups, exercise groups)

*Elaborated by the authors (2019). RECs, Specialized Rehabilitation Centers; HC, Health Center*.

## Conclusion

This study aimed to develop a referral flowchart based on the risk of falls for people with PD at the Brazilian PC. Brazil does not require mandatory reporting of PD. However, the Brazilian population of 60 years of age and over (approx. 28 million) and the increasing life expectancy of Brazilians suggest that PD and resulting falls are a significant health issue for older people.

We suggest that this flowchart might be the first step to build a multidisciplinary approach for people with PD in PC in Brazil. The next stage of this study might be the validation and subsequent implementation of the flowchart through PC at the UHS in Brazil.

### Limitations and future directions

Although prevention and management of falls of people with PD emerge in the qualitative research, we did not address specific questions for the FHS teams in the qualitative interview about this subject. Another limitation of our study was that we did not address the cognitive issues of people with PD. And this can influence the rate of falls in this population, so this must be considered when using the flowchart.

Also, the quality and quantity of the services offered at the PC varies among Brazilian regions. Like any standard, this flowchart cannot be taken as an absolute character, even if it applies to many situations. Therefore, a pilot study is needed for further improvements and to warrant the implementation in different regions.

As future directions, continuing education programs must be offered to health professionals at Primary Care. These professionals are the first contact with the PD patients through the Brazilian Health System. Hence, knowing how to identify, treat, and refer a person with PD could improve the quality of care and potentially increase the quality of life of those who live with the disease. Also, to use the flowchart its validation must occur, so the next step of this study might be its validation through the Primary Care at Unified Health System in Brazil.

## Data Availability Statement

The raw data supporting the conclusions of this manuscipt will be made available by the authors, without undue reservation.

## Ethics Statement

The studies involving human participants were reviewed and approved by Ethics Committee for Human Beings of the State University of Santa Catarina. The patients/participants provided their written informed consent to participate in this study.

## Author contributions

RSM, ECG, AS, and MHALK contributed to conception and design of the study. RSM organized the database, performed the statistical analysis, and wrote the first draft of the manuscript. AS and MHLAK revised the manuscript. All authors contributed to manuscript revision, read, and approved the submitted version.

## Funding

This study was financed in part by the Coordenação de Aperfeiçoamento de Pessoal de Nível Superior - Brasil (CAPES) - Finance Code 001.

## Conflict of interest

The authors declare that the research was conducted in the absence of any commercial or financial relationships that could be construed as a potential conflict of interest.

## Publisher's note

All claims expressed in this article are solely those of the authors and do not necessarily represent those of their affiliated organizations, or those of the publisher, the editors and the reviewers. Any product that may be evaluated in this article, or claim that may be made by its manufacturer, is not guaranteed or endorsed by the publisher.
